# Oregano Oil Emulsion Coatings as a Natural Approach to Extend the Shelf Life of Atlantic Bonito (*Sarda sarda*) Fillets

**DOI:** 10.3390/foods15162905

**Published:** 2026-08-19

**Authors:** Fernanda L. Ludtke, Jorge M. Vieira, Ítala Marx, António A. Vicente, Joana T. Martins

**Affiliations:** 1 CEB—Centre of Biological Engineering, University of Minho, 4710-057 Braga, Portugal; jorgevieira@ceb.uminho.pt (J.M.V.); itala.gouveia.marx@uco.es (Í.M.); avicente@deqb.uminho.pt (A.A.V.); joanamartins@ceb.uminho.pt (J.T.M.); 2 LABBELS—Associate Laboratory, University of Minho, 4710-057 Braga, Portugal

**Keywords:** edible coatings, fish products, essential oil, lipid oxidation, shelf-life evaluation

## Abstract

Atlantic bonito (*Sarda sarda*) is a commercially important fish species with high nutritional value that is widely harvested along the Portuguese coast; however, it is highly susceptible to lipid oxidation and microbial deterioration during refrigerated storage. This study investigated the effectiveness of oregano oil (OO) emulsion coatings, with and without fish gelatin, to preserve quality and increase shelf life of Atlantic bonito fillets stored at 5 °C for 10 days. Emulsions were produced by high-intensity ultrasound treatment and subsequently, applied as surface coatings. Physicochemical, microbiological, and quality-related parameters, including pH, water activity, lipid oxidation, protein oxidation, color, and texture, were evaluated throughout storage. Water activity of the coated fillets remained stable (*p* > 0.05), whereas pH values remained below 6.0 until day 7. Coated fillets exhibited reduced oxidative deterioration and delayed microbial growth compared with uncoated fillets, with the 10% OO emulsion coating (OO_10_) showing the higher effectiveness in maintaining color stability and limiting oxidation. The OO_10_ emulsion coating with fish gelatin (FO_10_) improved antimicrobial performance, although the gelatin matrix appeared to modulate the release of bioactive compounds from OO. Overall, OO emulsion coatings represent a promising natural preservation approach for extending the shelf life and maintaining the quality attributes of refrigerated fish products.

## 1. Introduction

Fish and fishery products are highly valued for their nutritional benefits, particularly due to their high content of essential amino acids, vitamins, and long-chain polyunsaturated fatty acids [1]. However, their high lipid and protein contents also make them highly susceptible to oxidative degradation and microbial spoilage after harvest, leading to rapid quality deterioration and a limited shelf life during refrigerated storage [2,3]. Among commercially important marine species, Atlantic bonito (*Sarda sarda*) is abundantly harvested along the Portuguese coast [4]. Despite its nutritional and economic importance, its rapid post-mortem deterioration remains a major challenge for the seafood industry.

Conventional preservation strategies frequently rely on synthetic additives; however, growing consumer demand for natural, sustainable, and clean-label products has encouraged the development of alternative preservation technologies [5,6,7]. Among these, edible coatings incorporating essential oils (EOs) have attracted considerable attention because of their ability to preserve the physicochemical and microbiological quality of fish fillets while extending shelf life [2,8].

EOs are recognized as promising natural preservatives owing to their antimicrobial and antioxidant activities. Oregano essential oil (OO), in particular, is rich in phenolic compounds such as carvacrol and thymol, which exhibit broad-spectrum antimicrobial activity and strong antioxidant capacity [9,10]. However, the direct incorporation of EOs into food systems is limited by their low water solubility, high volatility, and intense aroma, which may compromise their stability and sensory acceptability [2,11].

To overcome these limitations, emulsion-based delivery systems have been proposed to improve the dispersion, stability, and controlled release of EOs in food matrices [1,3,11].

In addition, the incorporation of biopolymers such as proteins into emulsion-based coatings may further enhance film-forming ability, adhesion to the food surface, and the controlled release of bioactive compounds, thereby improving emulsion-based coating performance during storage [2,12]. Among the proteins used on coatings’ production, gelatin has emerged as promising alternative due to its excellent film-forming and emulsifying properties, biocompatibility, and biodegradability. Particularly, gelatin obtained from fish-processing by-products is an attractive matrix for food preservation applications which promotes the valorization of marine biomass, contributing to the circular economy strategies [13].

Several studies have investigated the application of EO-based delivery systems as natural strategies to improve the preservation of fish and seafood products. For example, Hao et al. (2022) developed a stable EO-emulsion-based alginate coating system and demonstrated its potential as an effective green strategy for the preservation of refrigerated carp fillets [5]. Similarly, Shokri et al. (2020) reported that a chitosan coating incorporating *Ferulago angulata* essential oil effectively extended the shelf life of rainbow trout fillets [14]. More recently, Yang et al. (2024) developed and applied oregano/litsea cubeba essential oil nanoemulsion coatings to grass carp fillets, highlighting the potential of nanoemulsified EOs for controlling quality deterioration during refrigerated storage [15]. Other innovative EO delivery systems have also been explored, such as the oregano oil–lauric acid cationic nanostructured lipid carriers developed by Kumanbut et al. (2026), whose antibacterial activity and safety were evaluated in Nile tilapia [10]. In addition, Sayadi et al. (2025) developed edible Persian gum–gelatin films (1:1) incorporating thyme (*Zataria multiflora* Boiss) essential oil Pickering emulsions, further demonstrating the potential of biopolymer-based systems for the controlled incorporation of bioactive compounds into food packaging materials [8].

Despite these advances, limited information is available on the application of oregano oil emulsion coatings specifically for Atlantic bonito (*Sarda sarda*) fillets. Moreover, the combined use of fish gelatin and the evaluation of both simple and gelatin-stabilized oregano oil emulsions, including the effect of ultrasonication, remains insufficiently explored. Therefore, the present study aimed to evaluate the effects of OO emulsion coatings (either with or without fish gelatin), on the physicochemical, microbiological, and quality attributes of Atlantic bonito fillets during refrigerated storage, with particular emphasis on their ability to delay spoilage and extend shelf life.

## 2. Materials and Methods

### 2.1. Materials

Tween^®^ 80 (polysorbate 80) was purchased from Sigma-Aldrich (Saint Louis, MO, USA). Glycerol was obtained from BioChem Pharma (Oldenburg, Germany). Boric acid was supplied by Chem-Lab (Zedelgem, Belgium), while potassium carbonate and methyl red were purchased from Panreac (Barcelona, Spain). Hydrochloric acid was obtained from Labkem (Dublin, Ireland). Butylated hydroxytoluene (BHT), trichloroacetic acid (TCA), and 2-thiobarbituric acid (TBA) were acquired from Merck (Darmstadt, Germany). Fish gelatin was purchased from Sigma-Aldrich (Saint Louis, MO, USA). Oregano essential oil (OO) was supplied by Nutrifoods (Lliçà de Vall, Barcelona, Spain).

### 2.2. Emulsion’s Preparation and Characterization

In the first stage of the study, the emulsions were prepared using lipid phase consisting of sunflower oil supplemented with OO at concentrations of 10% (OO_10_), 20% (OO_20_) or 30% (OO_30_), together with 2% Tween 80 as emulsifier and 88% ultrapure water. The emulsions were produced following the method described by Sousa et al. (2024), with minor modifications [16]. Briefly, the lipid phase was gradually added dropwise to the aqueous phase containing the emulsifier while being homogenized with an Ultra-Turrax homogenizer (T18, Ika-Werke, Staufen im Breisgau, Germany) at 5000 rpm. The homogenization speed was subsequently increased to 7000 rpm and maintained for 3 min to obtain a coarse emulsion. The resulting pre-emulsion was then subjected to high-intensity ultrasonication (US) using a titanium microtip probe (13 mm diameter, 20 kHz; Vibra-Cell VCX 500, Sonics & Materials, Newtown, CT, USA) operated at 40% amplitude for 5 min in pulse mode (4 s on, 2 s off). Following the US treatment, the OO emulsion coatings containing fish gelatin (FO) were prepared by heating the emulsion to 45 °C, and fish gelatin (3%, *w*/*w*) was added under continuous stirring for 15 min. Subsequently, glycerol (0.9%, *w*/*w*) was incorporated, and the mixture was stirred for an additional 15 min. After preparation, the emulsion coatings were stored at 5 °C until further characterization and/or application.

#### 2.2.1. Droplet Size, Polydispersity Index and Zeta Potential

The mean droplet size (DS), polydispersity index (PDI), and zeta potential (ZP) of the emulsions were determined by dynamic light scattering (DLS) using a Zetasizer Nano ZS (Malvern Instruments, Worcestershire, UK) at 25 °C as reported by Lüdtke et al. (2023) [17]. For the OO-based emulsions (OO_10_, OO_20_ and OO_30_), measurements were performed 24 h after preparation and after 7, 14, 21, and 30 days of storage to evaluate physicochemical stability. The OO_10_ and OO_10_ emulsion coating with fish gelatin (FO_10_) formulations selected for application as edible coatings were characterized only 24 h after preparation. Prior to analysis, all samples were diluted 1:100 (*v*/*v*) with ultrapure water to minimize multiple scattering effects. DS was reported as the z-average diameter (nm).

#### 2.2.2. Contact Angle

Contact angle measurements were performed on the surface of Atlantic bonito fillets using an optical contact angle goniometer (OCA 20, DataPhysics, Filderstadt, Germany) equipped with a digital camera and image analysis software. At 25 °C, a 2 μL droplet of the coating formulation was carefully deposited onto the fillet surface using a 500 μL Hamilton microsyringe (Hamilton, Gräfelfing, Germany) at a controlled dispensing rate of one droplet per second [6]. The contact angle was then determined from the droplet profile using the instrument’s image-processing software.

### 2.3. Emulsions-Based Coatings Application

Atlantic bonito was harvested from the northern coast of Portugal, filleted by GuimarPeixe S.A. (Guimarães, Portugal), and transported to the laboratory under refrigerated conditions. The fillets (approximately 100 g each; ~12 cm × 9 cm × 2.5 cm) were immersed in the OO-based emulsion coatings (OO_10_ and/or FO_10_) for 5 s, followed by air-drying for 30 s in a laminar flow cabinet. Uncoated fillets served as the control treatment. Both coated and uncoated samples were individually packaged in polyethylene bags and stored for 10 days under refrigerated conditions (5 °C). Quality analyses were performed on storage days 0 (T0), 3 (T3), 5 (T5), 7 (T7), and 10 (T10).

### 2.4. Shelf-Life Evaluation

#### 2.4.1. Microbiological Characterization

For microbiological analyses, 25 g of each fish fillet sample was aseptically collected, homogenized with the appropriate diluent, and stomached for 2 min at room temperature. Serial 10-fold dilutions were subsequently prepared for microbial enumeration. Microbiological analyses of the control and coated fillets included the enumeration of *Enterobacteriaceae* on Violet Red Bile Dextrose agar following incubation at 37 °C for 48 h, according to ISO 21528-2:2017 [18], and total viable counts (TVC) on Tryptone Glucose Extract agar after incubation at 30 °C for 48 h, following ISO 4833-1:2013/Amd1:2022 [19]. All analyses were performed in triplicate for each treatment. Fish samples were collected from different regions of each Atlantic bonito fillet prior to homogenization to ensure representative sampling.

#### 2.4.2. pH Value and Water Activity

The pH of coated and uncoated Atlantic bonito fillets was determined by homogenizing 4 g of sample with 40 mL of distilled water using a vortex mixer. The pH was then measured using a digital pH meter (Hanna Instruments, Nușfalău, Romania) equipped with a surface electrode. Water activity (*a_W_*) was determined at 25 °C using an Aqualab 3 water activity meter (Decagon Devices, Pullman, WA, USA).

#### 2.4.3. Lipid Oxidation

Lipid oxidation was evaluated by determining thiobarbituric acid reactive substances (TBARS) according to the method described by Mendes et al. (2023) [6]. Absorbance was measured at 531 nm using a Synergy™ HT Multi-Detection Microplate Reader (BioTek Instruments, Inc., Winooski, VT, USA) with 96-well microplates. All analyses were performed in triplicate, and the results were expressed as mg of malondialdehyde (MDA) per kg^−1^ of sample.

#### 2.4.4. Total Volatile Basic Nitrogen (TVB-N)

The TVB-N content of Atlantic bonito fillets was determined using the Conway microdiffusion technique, following the methodology reported by Lüdtke et al. (2025) [4], with minor adaptations. Initially, 1 mL of 0.02% boric acid and 200 µL of a mixed indicator solution containing methyl red (0.001%) and bromocresol green (0.001%) in a 1:5 (*v*/*v*) ratio were dispensed into the inner compartment of a Conway dish. Subsequently, 1 mL of saturated potassium carbonate (K_2_CO_3_) solution and 1 mL of the filtrate, obtained by homogenizing Atlantic bonito fillets with distilled water followed by filtration through filter paper, were placed in the outer compartment. The Conway dish was immediately sealed to prevent ammonia loss and incubated at 25 °C for 90 min, allowing volatile nitrogenous compounds to diffuse into the boric acid solution. Following incubation, the boric acid solution was titrated with 0.01 mol L^−1^ HCl until the endpoint was indicated by the appearance of a pale pink color. All determinations were carried out in triplicate. The TVB-N content was expressed as mg N per 100 g of sample and calculated according to Equation (1) [20]:

*TVB-N* = (*V_1_* − *V_2_*) × *c* × 14 × (100/*m*) × (*V/V_0_*) (1)

where *V_1_* and *V_2_* correspond to the volumes (mL) of HCl required to titrate the sample and blank, respectively; *c* is the concentration (mol L^−1^) of the hydrochloric acid solution; 14 is the atomic mass of nitrogen (g mol^−1^); *m* is the mass of the fish sample (g); *V* is the volume of filtrate used in the analysis (mL); and *V_0_* represents the total volume of the filtrate (mL).

#### 2.4.5. Color Parameters

The effectiveness of the coating formulations in preserving the color of Atlantic bonito fillets during storage was evaluated by instrumental color analysis. Color measurements were performed using a colorimeter (CR-300 Chroma Meter, Minolta, Osaka, Japan). The color coordinates were determined according to the CIELAB color space, including L* (lightness), a* (redness/greenness), and b* (yellowness/blueness), for both coated and uncoated (control) fillets. For each replicate, three measurements were recorded at different locations on the fillet surface, and the mean values of L*, a*, and b* were used for statistical analysis. The overall color variation during storage was expressed as the total color difference (ΔE*), which was calculated according to Equation (2).

(2) $${\Delta E}^{*}=\sqrt{{\left({\Delta L}^{*}\right)}^{2}+{\left({∆a}^{*}\right)}^{2}+{\left({∆b}^{*}\right)}^{2}}$$

#### 2.4.6. Textural Properties

Texture profile analysis (TPA) was performed to characterize the textural properties of Atlantic bonito fillets by determining hardness, firmness, springiness, and gumminess. Measurements were carried out using a TA.HDPlus Texture Analyser (Stable Micro Systems Ltd., Godalming, UK) fitted with a 250 kg load cell, which generated force–time deformation curves during compression. The TPA consisted of a double-compression test in which samples were subjected to two consecutive compression cycles at a crosshead speed of 5 mm s^−1^. During the first cycle, the samples were compressed to 80% of their original height, followed by a second compression to 20% of the initial height after a 5 s recovery interval. The maximum compression force, the areas under the force–time curves for both compression cycles, and the distance from the onset of compression to the peak force were obtained from the force–time curves using Texture Exponent software (version 6.1.1.0, Stable Micro Systems, Surrey, UK). These parameters were subsequently used to calculate hardness, firmness, springiness, and gumminess. Atlantic bonito fillet samples were analyzed in triplicate at 21 °C.

### 2.5. Statistical Analysis

Statistical analyses were performed using OriginPro 2018^®^ software (OriginLab Corporation, Northampton, MA, USA). Data were analyzed by one-way analysis of variance (ANOVA), and mean comparisons were conducted using Tukey’s post hoc test at a significance level of 5% (*p* < 0.05). Results are presented as the mean ± standard deviation (SD).

## 3. Results and Discussion

### 3.1. Emulsions-Based Coatings Characterization

**Figure 1:**
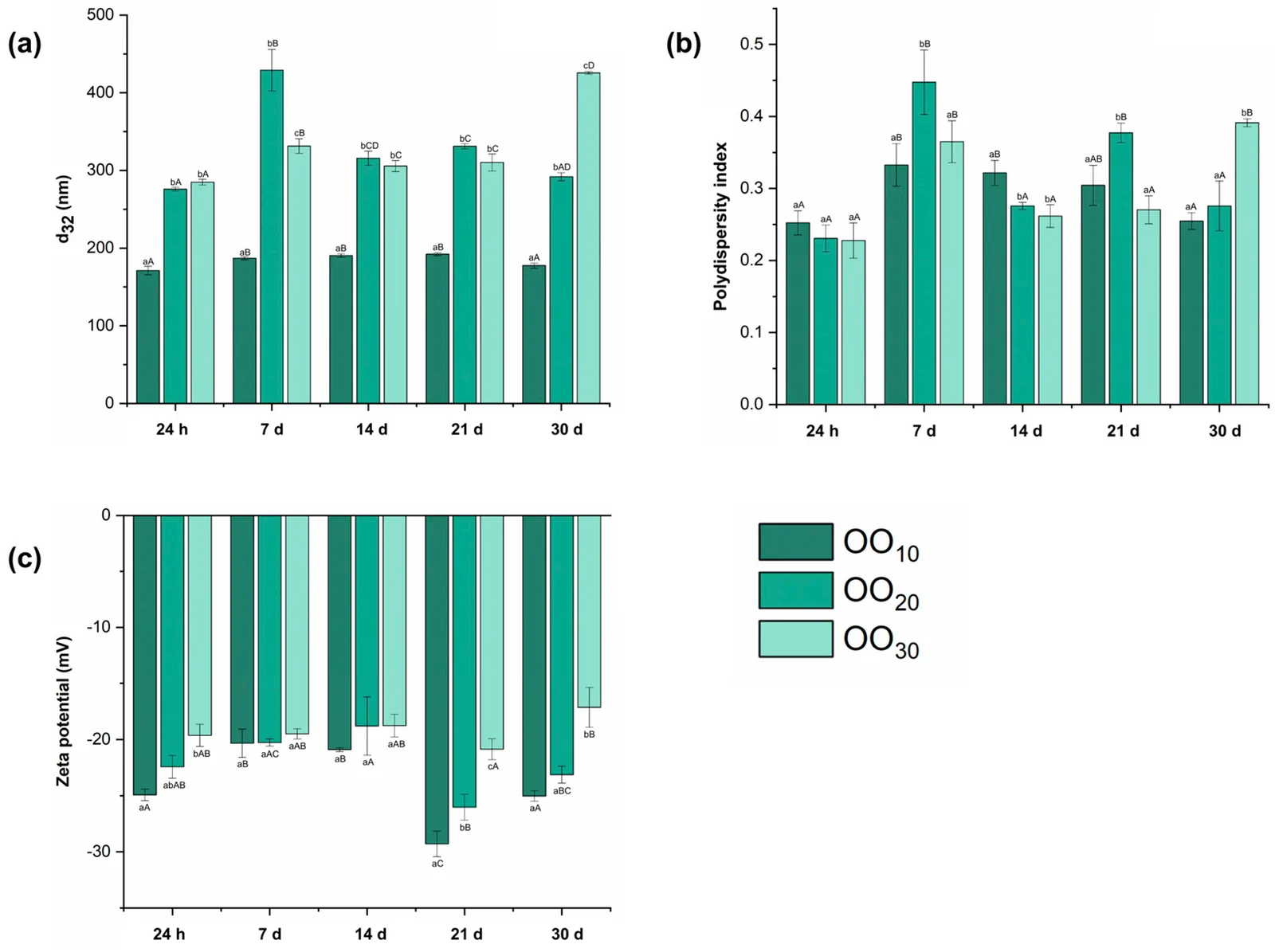
(**a**) Droplet size (d_32_), (**b**) polydispersity index, and (**c**) zeta potential (mV) of oregano oil-based emulsions (OO) at different concentrations (10, 20 and 30%). Error bars represent the standard deviation of *n* = 3 replicates. ^A–D^ Different capital letters indicate significant difference of the same sample during the storage time (*p* < 0.05). ^a–c^ Different lower-case letters indicate significant difference between samples in the same day of storage (*p* < 0.05).

To select the most stable emulsion for use as the coating formulation, the emulsions were characterized over a 30-day storage period by evaluating their DS, PDI, and ZP, as shown in Figure 1. The incorporation of different concentrations of OO into the lipid phase resulted in formulation-dependent effects on emulsion structure and stability during storage.

After 24 h, OO_10_ exhibited the smallest DS among the formulations, whereas OO_30_ showed the highest (171.10 ± 5.47 and 284.83 ± 3.72 nm, respectively), indicating that the increase in the OO proportion in the formulation promoted the formation of a coarser dispersion since the beginning of storage. Regarding the PDI, a significant increase was observed for the OO_30_ sample, which exhibited values above 0.39 ± 0.01 after 30 days of storage, whereas the OO_10_ and OO_20_ samples showed PDI values of 0.25 ± 0.01 and 0.28 ± 0.03, respectively, at the same time point. This finding indicates a reduction in structural uniformity in OO_30_ sample.

Throughout storage, OO formulations showed concentration-dependent behavior. The OO_10_ emulsion maintained relatively small DS and higher stability, whereas higher OO concentrations promoted structural rearrangements, with OO_20_ and OO_30_ showing increased DS at later storage stages. These findings are consistent with those of Ahmadi et al. (2026), who reported that increasing the essential oil (thyme oil) concentration from 2.5 to 20 mg.mL^−1^ progressively increased the mean DS (571.43–1042.37 nm) and the PDI (0.515–0.837), indicating that higher concentrations of phenolic compounds can adversely affect emulsion homogeneity [21].

The ZP results further supported the differences observed among the formulations. All emulsions exhibited negative ZP values, indicating the presence of electrostatic repulsion between droplets, which contributes to colloidal stability [22,23]. However, after 21 days of storage OO_10_ and OO_20_ samples consistently exhibited more negative ZP values than the OO_30_ sample, indicating greater electrostatic stabilization.

The higher OO content in the OO_30_ formulation may have influenced the interfacial organization, viscosity, and droplet–droplet interactions, thereby reducing the stability of this formulation [24]. Among all formulations, OO_10_ exhibited the most favorable physicochemical characteristics, combining the lowest DS, a narrow size distribution, and consistently negative ZP values, indicative of enhanced colloidal stability. Therefore, the OO_10_ formulation was selected as the base emulsion for application as an edible coating on Atlantic bonito fillets and for the development of the FO_10_.

Following the selection of the most suitable emulsion, the physicochemical characteristics of the coating formulations intended for application to Atlantic bonito fillets were further evaluated. Table 1 summarizes the DS, PDI, ZP, and contact angle of the OO_10_ and FO_10_ formulations. The incorporation of fish gelatin into the OO_10_ formulation modified the physicochemical properties of the emulsion, resulting in changes in DS, size distribution, surface charge, and wettability. These findings indicate that fish gelatin influenced the interactions among the emulsion components and altered the interfacial organization of the system.

The addition of fish gelatin increased the contact angle from 27.80° to 44.03°, indicating reduced surface wettability while maintaining the formulation within the hydrophilic range. This modification may influence coating performance by affecting spreading behavior, film formation, and adhesion to the fish surface.

A marked increase (*p* < 0.05) in DS was observed after gelatin incorporation, increasing from 177.70 nm in OO_10_ to 828.05 nm in FO_10_, together with an increase in PDI from 0.252 to 0.540. These differences indicate the formation of a more heterogeneous emulsion structure, which may influence the physical characteristics of the resulting coating, including its thickness, microstructure, and release behavior of OO. Additionally, the ZP value reduction from −24.93 mV in OO_10_ to 1.73 mV in FO_10_ reflects changes in the surface charge environment, suggesting that gelatin altered the balance of interactions governing droplet organization.

Overall, the incorporation of fish gelatin resulted in a distinct emulsion structure compared with OO_10_, modifying the physicochemical properties of the coating system. Although these changes do not necessarily indicate reduced functionality, they may influence coating performance by affecting film formation, adhesion to the fish fillet surface, barrier properties of the coating, and the release of OO during storage. Therefore, the interaction between OO and fish gelatin plays an important role in determining the final characteristics of the coating. Thus, the effects of applying these coating formulations to Atlantic bonito fillets and their influence on product quality during refrigerated storage are further discussed in the following sections.

### 3.2. Shelf-Life Evaluation

#### 3.2.1. Microbial Characterization

**Figure 2:**
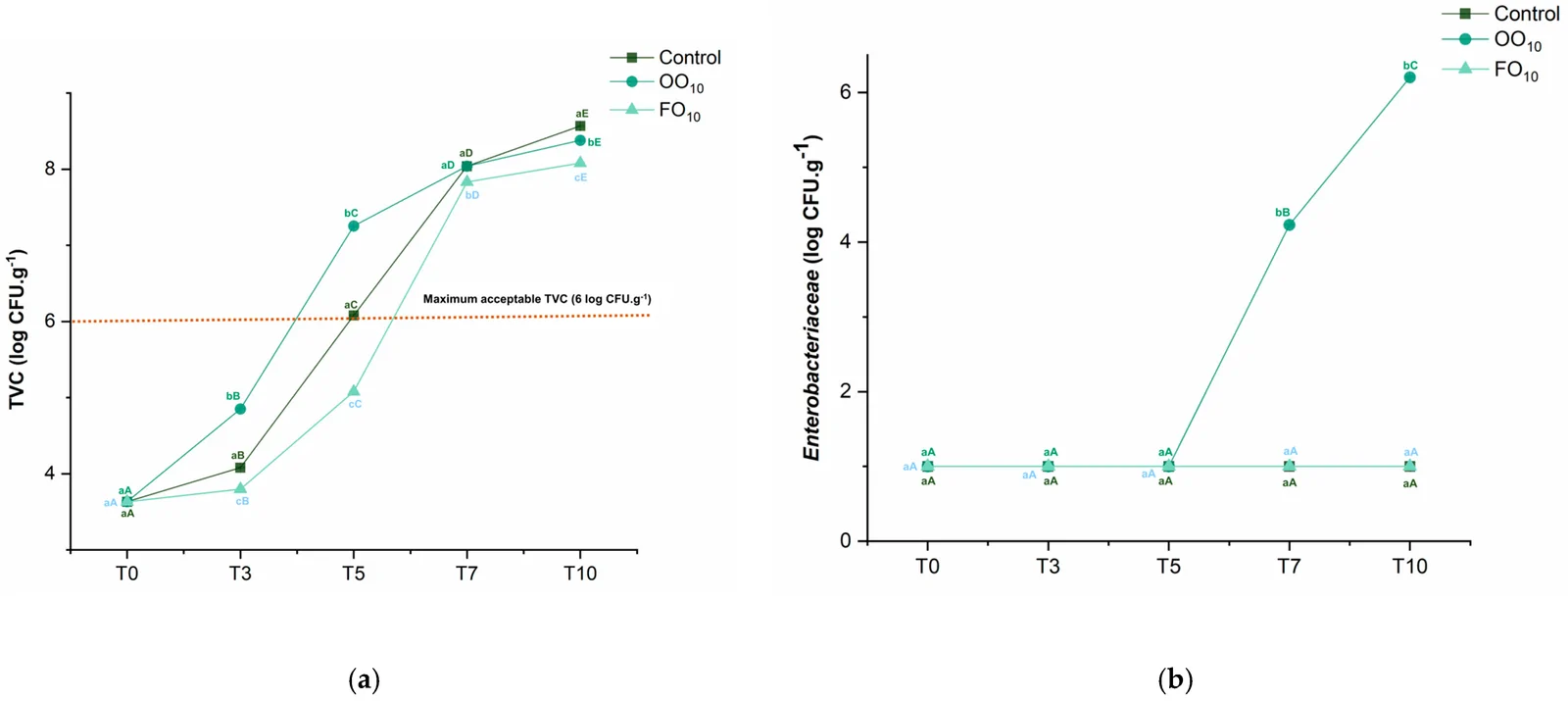
(**a**) Total viable count (TVC) and (**b**) *Enterobacteriaceae* counts of Atlantic bonito fillets samples (control, OO_10_ and FO_10_) over the storage period of 0 (T0), 3 (T3), 5 (T5), 7 (T7) and 10 (T10) days at 5 °C. Error bars represent the standard deviation of *n* = 3 replicates. ^A–E^ Different capital letters indicate significant difference of the same sample during the storage time (*p* < 0.05). ^a–c^ Different lower-case letters indicate significant difference between samples in the same day of storage (*p* < 0.05).

Figure 2 shows the effects of the coatings on microbial spoilage in refrigerated fillets, as evaluated by TVC and *Enterobacteriaceae* counts. The microbiological analyses revealed a progressive increase (*p* < 0.05) in the TVC throughout refrigerated storage for all samples. The uncoated control exhibited the highest microbial load on day 10, increasing from 3.63 log CFU.g^−1^ on day 0 to 8.57 log CFU.g^−1^, indicating rapid microbial spoilage in the absence of a protective coating.

Both OO-based coatings delayed microbial growth, particularly during the early stages of storage. The FO_10_ sample exhibited the highest inhibitory effect, with TVC values of 3.80 log CFU.g^−1^ on day 3 and 5.08 log CFU.g^−1^ on day 5, whereas the OO_10_ samples reached 4.85 log CFU.g^−1^ and 7.26 log CFU.g^−1^ on the respective sampling days. However, a marked increase (*p* < 0.05) in microbial counts was observed in both coated samples on days 7 and 10, indicating that the antimicrobial efficacy of the coatings gradually declined during storage.

TVC between 2 and 6 log CFU.g^−1^ are generally considered acceptable for maintaining the freshness of fishery products [25]. Accordingly, among the coated samples, the OO_10_ sample maintained acceptable microbiological quality up to day 3 (4.85 log CFU.g^−1^), whereas the FO_10_ sample remained within the acceptable limit until day 5 (5.08 log CFU.g^−1^), indicating a greater ability of the latter to delay microbial spoilage during refrigerated storage. Among the samples, the OO_10_ treatment presented the highest TVC values throughout storage. This may be partly attributable to differences in the initial microbial load rather than solely to the effect of the coating treatment. Variability in the initial microbiological quality of raw fish can arise from differences in the level of microbial contamination, handling practices, and post-harvest conditions.

The *Enterobacteriaceae* counts remained below the detection limit (<10 CFU.g^−1^) in most samples throughout storage, with detectable levels observed only in OO_10_ on days 7 and 10. The exclusive detection of *Enterobacteriaceae* in OO_10_ is likely attributable to a low-level initial contamination originated from the raw materials and/or introduced during sample preparation and processing. Since *Enterobacteriaceae* remained below the detection limit in all other samples throughout storage, these findings suggested that the contamination was localized rather than widespread. A low initial microbial load may become detectable during storage if the formulation provides favorable conditions for microbial survival. Therefore, the detected *Enterobacteriaceae* in OO_10_ most likely originated from residual microorganisms associated with the fish, processing equipment, or handling procedures, rather than indicating a general microbiological instability of the coating system. Nevertheless, the counts observed on the final storage day in the present study were lower than those reported by Maghsoudi et al. (2025), who found 6.22 and 4.91 log CFU.g^−1^ after 7 days of storage in rainbow trout fillets coated with a mucilage coating containing 2% *Satureja hortensis* EO and a mucilage coating containing a 2% nanoemulsion of *S. hortensis* EO, respectively [1]. Similarly, Sayadi et al. (2025) reported *Enterobacteriaceae* counts of 4.34 log CFU.g^−1^ after 6 days of refrigerated storage in barred mackerel fillets coated with a Persian gum–gelatin Pickering emulsion film loaded with thyme oil [8]. These comparisons indicate that, despite the localized detection of *Enterobacteriaceae* in OO_10_, microbial levels remained relatively low compared with those reported for other active coating systems. The *Enterobacteriaceae* results further highlight the superior antimicrobial performance of the FO_10_ formulation. Although fish gelatin does not possess intrinsic antimicrobial activity, its incorporation as a film-forming matrix likely improved the structural integrity and adhesion of the coating, thereby enhancing its barrier properties and limiting oxygen and moisture transfer at the fish surface [13]. Moreover, the gelatin matrix may have promoted a more controlled release of the bioactive compounds present in OO by stabilizing the emulsion and reducing the rapid diffusion of phenolic constituents, such as carvacrol and thymol. Consequently, despite OO_10_ formulation containing the same concentration of OO as the FO formulation, the gelatin-based coating exhibited a more sustained antimicrobial effect during refrigerated storage, effectively suppressing the growth of *Enterobacteriaceae* throughout most of the storage period and maintaining the microbiological freshness of the fillets for up to 5 days under refrigerated conditions.

#### 3.2.2. pH Value and Water Activity

All samples presented *a_W_* values close to 0.98 throughout the 10-day storage period, with no significant differences (*p* > 0.05) among the samples, indicating that the edible coatings did not affect the internal water status of the fillets.

**Figure 3:**
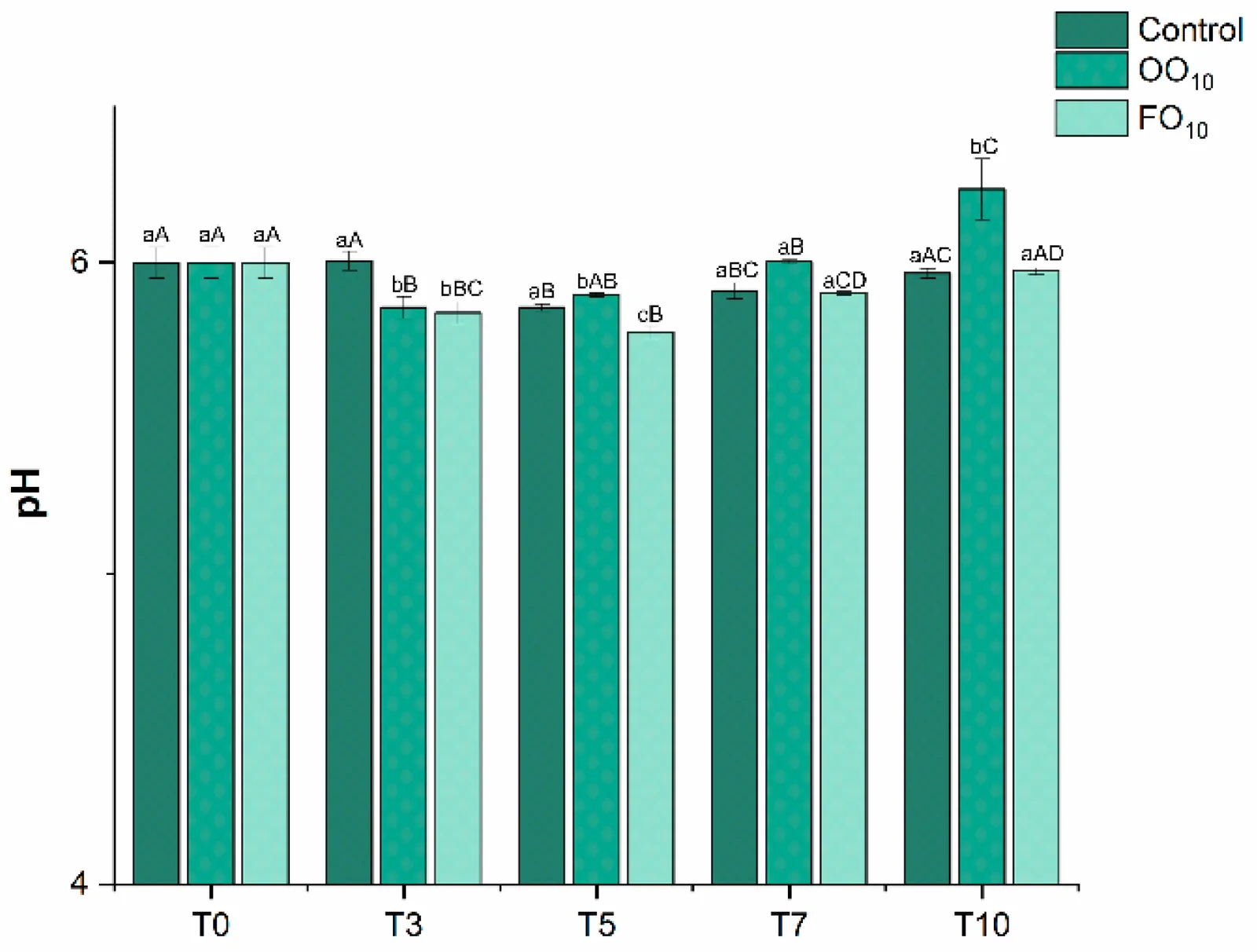
pH value of Atlantic bonito fillets samples (control, OO_10_ and FO) over the storage period of 0 (T0), 3 (T3), 5 (T5), 7 (T7) and 10 (T10) days at 5 °C. Error bars represent the standard deviation of *n* = 3 replicates. ^A–D^ Different capital letters indicate significant difference of the same sample during the storage time (*p* < 0.05). ^a–c^ Different lower-case letters indicate significant difference between samples in the same day of storage (*p* < 0.05).

Figure 3 presents the pH values of bonito fillets during refrigerated storage. All samples exhibited an initial pH of approximately 6.0, which is characteristic of fresh fish muscle [26]. During storage, only minor pH fluctuations were observed, reflecting the balance between the formation of acidic metabolites and the accumulation of alkaline compounds associated with microbial activity and protein degradation [21]. The control sample showed relatively stable pH values throughout storage, with a slight decrease to 5.86 on day 5 followed by a gradual increase to 5.97 on day 10. A similar trend was observed for the OO_10_ samples, which reached its lowest pH (5.86) on day 3 before increasing to 6.24 on day 10. The higher final pH in OO_10_ sample suggests greater accumulation of alkaline metabolites during the later stages of storage, which may be associated with the microbial growth observed in OO_10_ sample during the later stages of storage (Section 3.2.1).

In contrast, the FO_10_ sample exhibited the lowest pH value on day 5 (5.78) and maintained a more stable pH profile throughout storage, reaching 5.97 on day 10. This behavior indicates a slower progression of biochemical and microbial changes compared with OO_10_. The improved pH stability may be attributed to the incorporation of fish gelatin, which likely enhanced the barrier properties of the coating and promoted a more sustained release of the bioactive compounds present in OO.

The obtained results indicate that OO-based coatings moderated pH changes during refrigerated storage, while the incorporation of fish gelatin further improved coating performance by maintaining pH stability and delaying spoilage-related changes in Atlantic bonito fillets.

#### 3.2.3. Lipid Oxidation

Lipid oxidation was assessed by the TBARS assay, one of the most widely used methods for evaluating secondary lipid oxidation in fish and fishery products. This assay quantifies malondialdehyde (MDA), together with other aldehydic compounds formed during the oxidative degradation of polyunsaturated fatty acids [4]. The high content of unsaturated lipids in marine fish makes them particularly prone to oxidative deterioration, compromising product quality.

**Figure 4:**
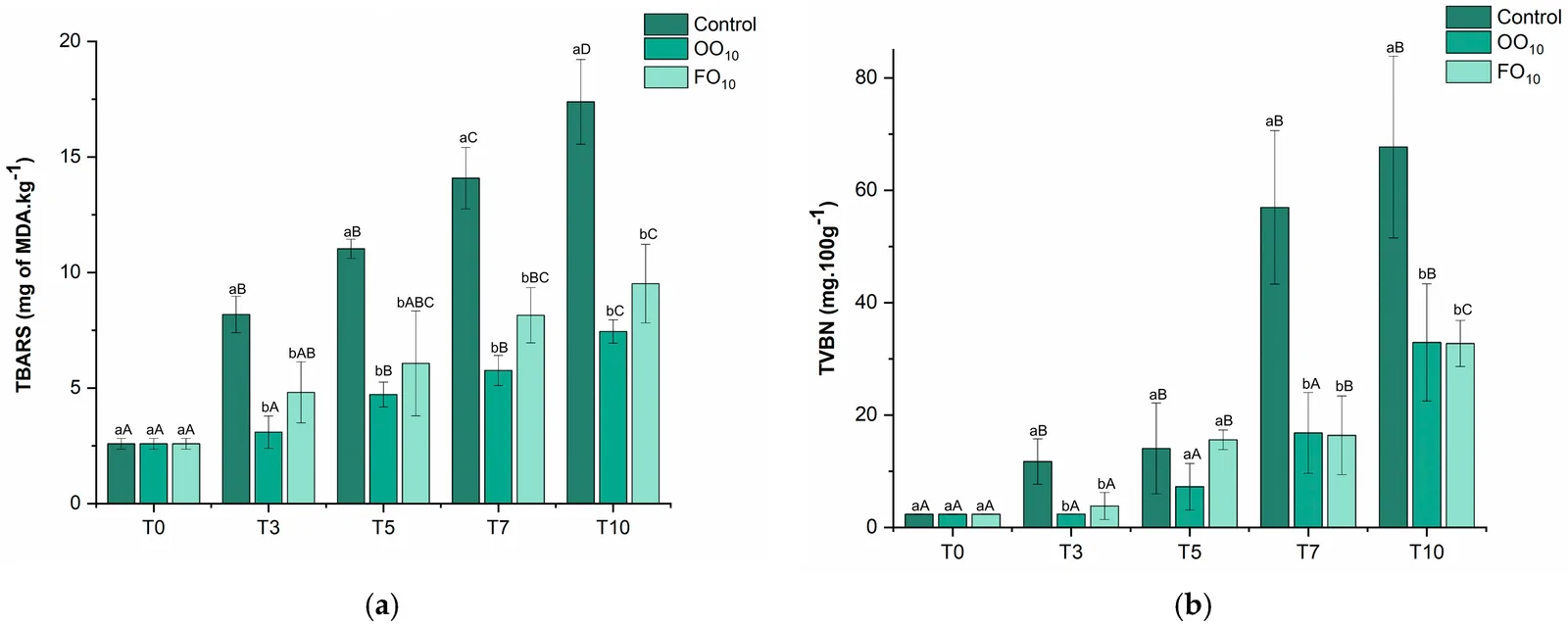
(**a**) Thiobarbituric acid reactive substances (TBARS) (mg of malonaldehyde (MDA).kg^−1^) and (**b**) Total Volatile Base Nitrogen (TVB-N) (mg.100 g^−1^) of Atlantic bonito fillets samples (control, OO_10_ and FO_10_) over the storage period of 0 (T0), 3 (T3), 5 (T5), 7 (T7) and 10 (T10) days at 5 °C. Error bars represent the standard deviation of *n* = 3 replicates. ^A–D^ Different capital letters indicate significant difference of the same sample during the storage time (*p* < 0.05). ^a,b^ Different lower-case letters indicate significant difference between samples in the same day of storage (*p* < 0.05).

Figure 4a illustrates the changes in TBARS values of the control and coated fillets during refrigerated storage. The control samples exhibited the highest TBARS values throughout storage, increasing from 2.58 to 17.39 on day 0 to day 10, respectively, indicating extensive lipid oxidation in the absence of a protective coating. The TBARS values observed in the control samples in the present study were considerably higher than those reported in several previous studies. For instance, Vital et al. (2018) reported a TBARS value of 0.41 ± 0.02 mg MDA/kg at the end of 14 days of refrigerated storage in tilapia (*Oreochromis niloticus*) [27]. Similarly, Ruelas-Chacón et al. (2020) reported a value of 0.54 ± 0.90 mg MDA/kg after 15 days of storage in tilapia (*Oreochromis niloticus*) fillets [28]. In contrast, Kanelaki et al. (2022) reported TBARS values approaching 40 mg MDA/kg in uncoated fresh sardine fillets [29]. The considerable variation in TBARS values reported among studies may be attributed to several factors, including differences in fish species, fishing conditions, and post-harvest handling and treatment of the samples, all of which can influence lipid oxidation and the formation of secondary oxidation products.

Both OO_10_ and FO_10_ coatings significantly inhibited lipid oxidation compared with the control. Among the samples, OO_10_ was the most effective, exhibiting the lowest TBARS values throughout storage and reaching only 7.44 mg MDA.kg^−1^ on day 10. This pronounced antioxidant effect is likely associated with the phenolic constituents of OO, particularly carvacrol and thymol, which act as free radical scavengers and inhibit the formation of secondary lipid oxidation products [2,30].

The FO_10_ also reduced lipid oxidation, with TBARS values reaching 9.52 mg MDA.kg^−1^ on day 10. Although significantly lower (*p* < 0.05) than those of the control, these values remained consistently higher than those observed for OO_10_. As both coatings contained the same concentration of OO, the lower antioxidant efficacy of FO_10_ suggests that the fish gelatin matrix limited the release and availability of phenolic compounds, likely through protein–phenolic interactions. Although the oxygen barrier properties of gelatin still contributed to reducing lipid oxidation, this effect was insufficient to compensate for the reduced antioxidant activity, resulting in higher TBARS values than in OO_10_. Similar to our findings, low TBARS values have been reported in fish products treated with coatings containing EOs. For instance, Lambrianidi et al. (2019) demonstrated that eel fillets coated with chitosan and OO and stored under vacuum packaging exhibited significantly lower concentrations of TBARS compared with the control samples [12]. Likewise, Chuesiang et al. (2020) reported that the application of a cinnamon oil nanoemulsion to Asian seabass effectively delayed lipid oxidation during storage [11]. Sayadi et al. (2025) further observed that Persian gum-based edible films loaded with TO Pickering emulsions delayed lipid oxidation in barred mackerel fillets [8]. In addition, Ahmadi et al. (2026) reported minimal lipid oxidation in rainbow trout coated with chitosan/nanoclay nanocomposite coatings enriched with thyme oil [21].

Considering the acceptable TBARS limit for fish of 5–8 mg MDA/kg reported by Mol et al. (2023) [31], the control samples exceeded this threshold after only 3 days of refrigerated storage, whereas the FO_10_ treatment exceeded it on day 7. In contrast, the OO_10_ treatment remained below 8 mg MDA/kg throughout the entire storage period. Collectively, these studies support the effectiveness of EO-based coatings and films in reducing lipid oxidation and improving the oxidative stability of fish products during storage.

#### 3.2.4. Total Volatile Basic Nitrogen (TVB-N)

TVB-N is a widely accepted indicator of fish freshness, reflecting the accumulation of volatile nitrogenous compounds, including ammonia, trimethylamine, and dimethylamine, produced through microbial activity and endogenous enzymatic degradation [32].

The TVB-N results, presented in Figure 4b, demonstrate that OO-based coatings effectively delayed protein degradation in bonito fillets during refrigerated storage compared with the uncoated control. TVB-N values increased progressively (*p* < 0.05) in all samples throughout storage, indicating gradual fish spoilage. However, the rate of increase differed markedly among samples. The control samples exhibited the highest TVB-N values, increasing from 2.34 mg N.100 g^−1^ on day 0 to 56.96 mg N.100 g^−1^ on day 7 and reaching 67.71 mg N.100 g^−1^ on day 10. This pronounced increase indicates extensive proteolytic degradation in the absence of a protective coating. In contrast, both OO-based coatings significantly inhibited the accumulation of volatile basic nitrogen compounds compared with the control. Among the samples, OO_10_ showed the greatest effectiveness, maintaining the lowest TVB-N values until day 5 of storage (7.23 mg N.100 g^−1^). The FO coating also reduced TVB-N accumulation compared with the control; however, its effectiveness was lower than that of OO_10_, particularly up to day 5 of storage. TVB-N values of FO_10_ sample increased to 15.59 mg N.100 g^−1^ on day 5 and reached 32.73 mg N.100 g^−1^ on day 10, a value comparable to that observed for OO_10_ at the same storage time.

As both coatings contained the same concentration of OO, the lower efficacy of FO_10_ suggests that the incorporation of fish gelatin influenced the release and diffusion of the bioactive compounds, thereby reducing their antioxidant effectiveness during the early stages of storage. Consistent with the TBARS results, the gelatin matrix may have limited the diffusion and availability of OO phenolic compounds through protein–phenolic interactions, thereby restricting their antioxidant activity at the fish surface. Although gelatin may have provided additional barrier properties, these were insufficient to compensate for the reduced accessibility of the active compounds. Furthermore, fish gelatin may have contributed to the higher TVB-N values observed in FO_10_ by serving as an additional nitrogen source susceptible to enzymatic degradation, thereby promoting the formation of volatile nitrogenous compounds during the initial storage period.

According to Rezaeifar et al. (2020), the acceptable limit of TVB-N for refrigerated fish products is 25 mg N·100 g^−1^ muscle [33]. In this context, both the OO_10_ and FO_10_ coatings were able to delay the accumulation of volatile nitrogenous compounds, maintaining TVB-N values below the acceptable limit until at least day 7 of storage. In contrast, the control samples exceeded 50 mg N·100 g^−1^ muscle by day 7, indicating a substantially faster deterioration process. Similar results have been reported for rainbow trout treated with *Zataria multiflora* essential oil combined with potassium sorbate [34], chilled common carp coated with alginate-based films containing 1% TO, OO, or pimento essential oil emulsions [5], and rainbow trout fillets coated with chitosan containing a moringa (*Moringa oleifera*) oil nanoemulsion [35]. These studies consistently demonstrate that EO-based coatings effectively retard the formation of volatile nitrogenous compounds, thereby preserving fish freshness and extending refrigerated shelf life.

#### 3.2.5. Colorimetric Parameters

**Figure 5:**
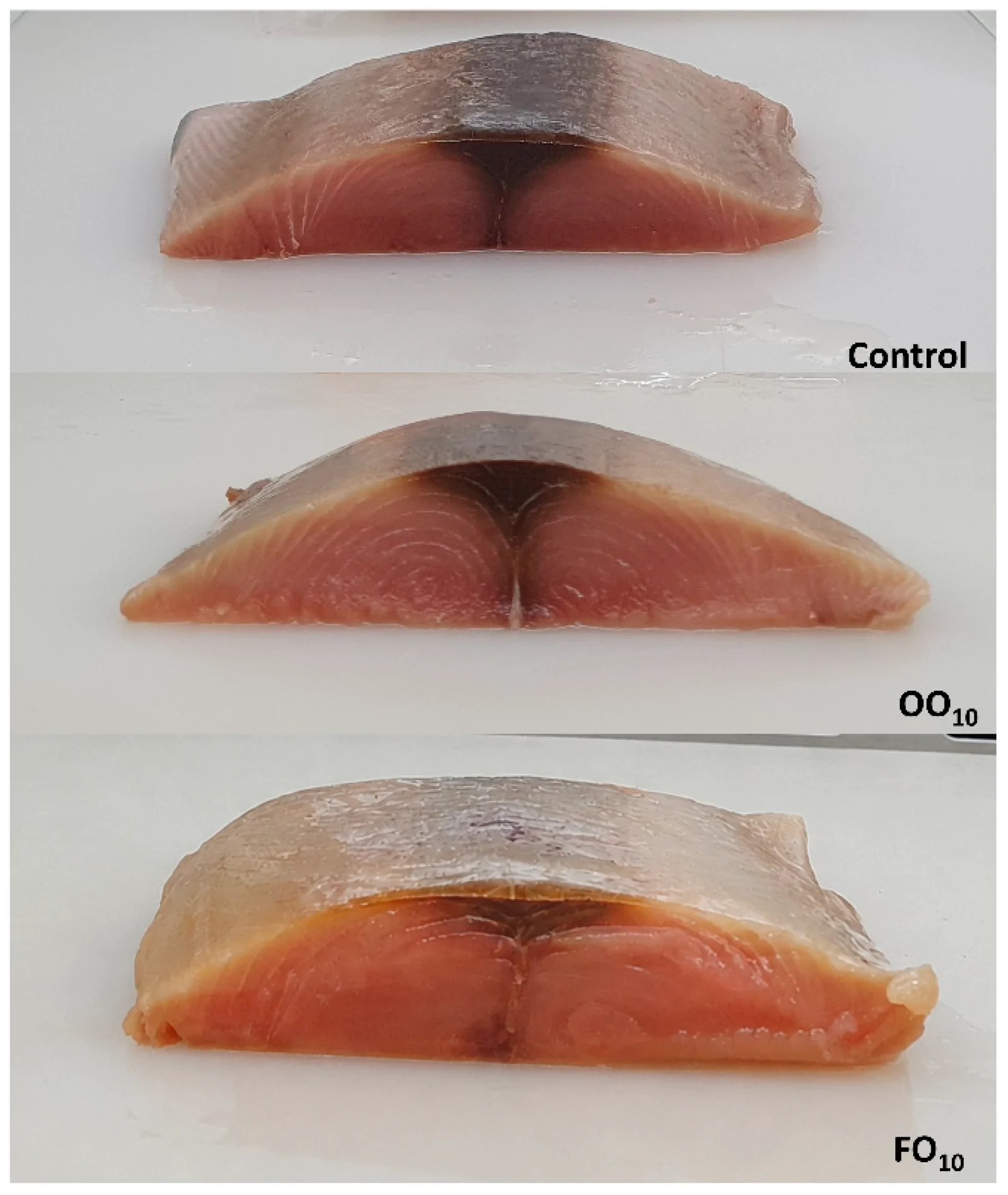
Visual appearance of Atlantic bonito fillets samples (control, OO_10_ and FO_10_) after 5 days of storage.

Figure 5 illustrates the visual appearance of the Atlantic bonito fillets after 5 days of storage. The color parameters of Atlantic bonito fillets were significantly influenced by both storage time and coating formulation (Table 2). The control samples exhibited a significant decrease in redness (a*) after 5 days of refrigerated storage, indicating pigment oxidation and progressive quality deterioration. The reduction in a* values is commonly associated with the oxidation of myoglobin and hemoglobin derivatives, leading to the formation of metmyoglobin and other oxidized pigments that impart a less appealing appearance to fish products [36]. In contrast, fillets treated with the FO_10_ coating maintained the highest a* value at the end of the storage period (6.08 ± 1.35), indicating superior preservation of the characteristic Atlantic bonito red color.

The yellowness parameter (b*) increased significantly after 3 days of refrigerated storage in all samples, with no significant differences (*p* > 0.05) observed among the coating formulations. The increase in b* values has been frequently associated with the accumulation of secondary lipid oxidation products and the degradation of natural pigments during refrigerated storage. As all samples exhibited a similar trend, the coatings appeared to have a limited effect on preventing the development of yellowish coloration, despite their beneficial effects on other quality attributes.

Lightness (L*) increased significantly (*p* < 0.05) from day 3 of refrigerated storage onward in all samples. However, at the end of the storage period, fillets treated with the OO_10_ coating exhibited a significantly lower L* value (52.62 ± 1.91) than the control samples, suggesting better preservation of the original appearance of the fillets. This effect may be attributed to the antioxidant properties of OO, which delayed the oxidative reactions responsible for muscle discoloration.

These observations were further supported by the total color difference (ΔE*), which provides a more comprehensive assessment of overall visual quality changes. The control samples exhibited a marked increase in ΔE* throughout refrigerated storage, reaching values above 17 after 10 days, indicating pronounced color deterioration. In contrast, the OO_10_ samples consistently exhibited lower ΔE* values, particularly on day 5 (ΔE* = 9.83), demonstrating that the OO coating effectively delayed visually perceptible color changes. This protective effect is consistent with the lower lipid oxidation observed in the TBARS analysis, reinforcing the antioxidant role of OO phenolic compounds in preserving pigment stability. The FO_10_ samples exhibited intermediate ΔE* values up to day 5 of storage. The relatively higher ΔE* observed during the early storage period may be attributed not only to oxidative changes but also to the optical properties of the gelatin-based coating, which can alter light scattering and surface reflectance independently of pigment degradation. Nevertheless, the lower ΔE* values observed for both coated samples compared with the control throughout storage indicate that the coatings contributed to preserving the visual quality of the fillets, with the OO_10_ coating showing the greatest effectiveness.

#### 3.2.6. Textural Properties

The evolution of the textural properties of Atlantic bonito fillets during refrigerated storage provides valuable insights into the effects of the coating formulations on muscle structural integrity and quality preservation. Table 3 summarizes the changes in the textural parameters of Atlantic bonito fillets throughout refrigerated storage under the different coating treatments.

No significant differences (*p* > 0.05) in hardness were observed among samples at day 0, indicating comparable initial conditions. Throughout refrigerated storage, hardness values remained stable in all samples, with only minor fluctuations. On day 5, fillets coated with OO_10_ exhibited a slight increase in hardness (14.39 ± 0.10 kg), whereas those treated with FO_10_ showed a lower value (11.35 ± 0.68 kg), suggesting that the fish gelatin-based coating may have promoted a softer texture, possibly as a result of enhanced moisture retention. However, these differences were not consistently maintained throughout storage, indicating that the coatings had only a limited long-term effect on hardness.

Similarly, springiness remained relatively constant throughout storage, with no significant differences (*p* > 0.05) among samples or days of storage. Values ranged from approximately 22% to 30%, indicating that the elastic properties of the muscle were largely preserved regardless of the coating formulation.

In contrast, fillets gumminess and chewiness exhibited higher changes during storage. Gumminess increased significantly (*p* < 0.05) during refrigerated storage in all samples, particularly between days 3 and 5, which may be associated with protein denaturation and the redistribution of water within the muscle matrix. Although no consistent significant differences were observed among samples, fillets coated with FO_10_ tended to exhibit lower chewiness values at specific sampling times, particularly on days 5 and 7. This trend may indicate that the gelatin-containing coating helped mitigate the progressive toughening of the muscle structure during storage.

Although textural parameters were modestly influenced by the coatings, the results suggest that both OO_10_ and FO_10_ contributed to maintaining fillets’ structural quality.

## 4. Conclusions

An increase in the OO concentration in the formulations resulted in increased DS and PDI, accompanied by lower ZP values, indicating reduced emulsion stability and a less homogeneous emulsion structure. The OO_10_ coating proved to be the most effective in preserving visual quality of Atlantic bonito fillet, maintaining color for up to 7 days and resulting in lower total color difference (ΔE*) values than the uncoated control. Although the FO_10_ coating also reduced ΔE* compared with the control, its color-preserving effect was maintained only until day 5.

The incorporation of fish gelatin into the coating formulation enhanced its antimicrobial performance, leading to lower microbial loads during refrigerated storage. However, the biopolymer matrix also appeared to delay the release of OO phenolic compounds, thereby reducing their immediate antioxidant activity and resulting in higher levels of lipid and protein oxidation than that observed for the OO_10_ formulation. These findings emphasize the importance to optimize the balance between barrier properties and the controlled release of bioactive compounds when developing active edible coatings.

Overall, the results indicated that OO emulsion-based edible coatings are a promising natural strategy for extending the refrigerated shelf life of Atlantic bonito fillets and preserving their quality. In particular, the OO_10_ formulation effectively maintained the color of the fillets and limited oxidative deterioration, whereas the incorporation of fish gelatin improved antimicrobial performance. Further studies are necessary to elucidate the release kinetics of bioactive compounds from FO matrix and to optimize coating formulations to maximize both antioxidant and antimicrobial effectiveness. Future studies should focus on elucidating the release kinetics of OO phenolic compounds from fish gelatin matrices and optimizing the balance between antioxidant protection and sustained antimicrobial activity.

## Figures and Tables

**Table 1:** Contact angle, droplet size (DS), polydispersity index (PDI) and zeta potential (ZP) of oregano oil emulsion coatings (OO_10_) and OO_10_ with fish gelatin (FO_10_).

Sample	Contact Angle (°)	DS (nm)	PDI	ZP (mV)
OO_10_	27.80 ± 6.40 ^a^	177.70 ± 5.47 ^a^	0.252 ± 0.02 ^a^	−24.93 ± 0.51 ^a^
FO_10_	44.03 ± 2.55 ^b^	828.05 ± 9.38 ^b^	0.540 ± 0.08 ^b^	1.73 ± 0.24 ^b^

**^a^**^,b^ Different lower-case letters indicate significant difference between coatings (*p* < 0.05).

**Table 2:** Color parameters obtained for control and coated Atlantic bonito fillets on days 0 (T0), 3 (T3), 5 (T5), 7 (T7) and 10 (T10) of storage.

Storage Time	Sample	L*	a*	b*	ΔE*
T0	Control	40.44 ± 1.74 ^aA^	6.67 ± 1.35 ^aA^	5.03 ± 0.62 ^aA^	-
	OO_10_	40.44 ± 1.74 ^aA^	6.67 ± 1.35 ^aA^	5.03 ± 0.62 ^aA^	-
	FO_10_	40.44 ± 1.74 ^aA^	6.67 ± 1.35 ^aA^	5.03 ± 0.62 ^aA^	-
T3	Control	52.13 ± 2.13 ^aB^	6.34 ± 2.41 ^aA^	9.50 ± 0.36 ^aB^	12.69 ± 2.00 ^aA^
	OO_10_	50.51 ± 1.13 ^aBC^	2.90 ± 0.45 ^aB^	8.86 ± 0.76 ^aB^	11.45 ± 0.87 ^aAB^
	FO_10_	52.28 ± 2.74 ^aB^	4.84 ± 0.49 ^aABC^	9.76 ± 0.63 ^aB^	14.80 ± 2.40 ^aA^
T5	Control	57.43 ± 1.31 ^aC^	1.20 ± 0.66 ^aB^	11.15 ± 3.37 ^aB^	19.00 ± 2.37 ^aB^
	OO_10_	48.24 ± 1.44 ^bB^	3.95 ± 0.56 ^bB^	10.29 ± 0.56 ^aBC^	9.83 ± 1.28 ^bA^
	FO_10_	54.23 ± 0.50 ^cB^	3.81 ± 0.34 ^bBC^	10.29 ± 0.41 ^aB^	15.04 ± 0.36 ^cA^
T7	Control	56.76 ± 2.14 ^aC^	3.13 ± 1.10 ^aAB^	10.64 ± 0.33 ^aB^	17.64 ± 2.14 ^aB^
	OO_10_	50.42 ± 1.37 ^bBC^	4.77 ± 0.98 ^aAB^	11.08 ± 0.75 ^aC^	11.89 ± 1.19 ^bAB^
	FO_10_	57.12 ± 2.16 ^aB^	2.63 ± 0.17 ^aB^	10.54 ± 0.75 ^aB^	15.06 ± 1.65 ^abA^
T10	Control	56.32 ± 0.29 ^aBC^	3.87 ± 0.19 ^aAB^	11.81 ± 0.17 ^aB^	17.49 ± 0.23 ^aAB^
	OO_10_	52.62 ± 1.91 ^bC^	3.73 ± 0.45 ^aB^	11.12 ± 1.01 ^aC^	13.95 ± 1.98 ^aB^
	FO_10_	54.12 ± 1.60 ^abB^	6.08 ± 1.35 ^bC^	11.19 ± 0.47 ^aB^	18.04 ± 2.17 ^aA^

^A–C^ Different capital letters indicate significant difference of the same sample during the storage time (*p* < 0.05). ^a–c^ Different lower-case letters indicate significant difference between samples in the same day of storage (*p* < 0.05).

**Table 3:** Textural parameters (hardness, springiness, gumminess and chewiness) obtained for control and coated Atlantic bonito fillets on days 0 (T0), 3 (T3), 5 (T5), 7 (T7) and 10 (T10) of storage.

Storage Time	Sample	Hardness (kg)	Springiness (%)	Gumminess	Chewiness
T0	Control	12.39 ± 0.95 ^aA^	24.47 ± 6.09 ^aA^	1.36 ± 0.15 ^aA^	0.34 ± 0.12 ^aA^
	OO_10_	12.39 ± 0.95 ^aA^	24.47 ± 6.09 ^aA^	1.36 ± 0.15 ^aA^	0.34 ± 0.12 ^aA^
	FO_10_	12.39 ± 0.95 ^aAB^	24.47 ± 6.09 ^aA^	1.36 ± 0.15 ^aA^	0.34 ± 0.12 ^aA^
T3	Control	12.43 ± 0.52 ^aA^	24.88 ± 4.43 ^aA^	2.00 ± 0.12 ^aB^	0.50 ± 0.11 ^aA^
	OO_10_	12.29 ± 0.45 ^aA^	25.78 ± 3.41 ^aA^	1.72 ± 0.24 ^aAB^	0.44 ± 0.05 ^aA^
	FO_10_	13.08 ± 0.13 ^aA^	30.52 ± 5.65 ^aA^	2.07 ± 0.16 ^aB^	0.64 ± 0.15 ^aA^
T5	Control	12.75 ± 0.50 ^aA^	26.07 ± 5.81 ^aA^	1.95 ± 0.03 ^aBC^	0.51 ± 0.12 ^aA^
	OO_10_	14.39 ± 0.10 ^abA^	24.25 ± 13.95 ^aA^	2.27 ± 0.57 ^aAB^	0.55 ± 0.50 ^aA^
	FO_10_	11.35 ± 0.68 ^bAB^	22.97 ± 4.68 ^aA^	1.82 ± 0.23 ^aAB^	0.43 ± 0.14 ^aA^
T7	Control	11.18 ± 0.72 ^aA^	26.19 ± 4.47 ^aA^	1.54 ± 0.21 ^aAC^	0.41 ± 0.10 ^aA^
	OO_10_	11.31 ± 0.48 ^aA^	26.88 ± 9.40 ^aA^	2.11 ± 0.58 ^aB^	0.58 ± 0.25 ^aA^
	FO_10_	11.17 ± 0.52 ^aB^	24.18 ± 5.85 ^Aa^	1.85 ± 0.29 ^aAB^	0.43 ± 0.03 ^aA^
T10	Control	11.48 ± 0.64 ^Aa^	27.06 ± 3.60 ^aA^	2.04 ± 0.27 ^aB^	0.55 ± 0.11 ^aA^
	OO_10_	12.06 ± 0.20 ^aA^	21.77 ± 4.33 ^aA^	1.89 ± 0.18 ^aAB^	0.41 ± 0.06 ^aA^
	FO_10_	11.95 ± 1.02 ^aAB^	23.52 ± 4.85 ^aA^	2.12 ± 0.27 ^aB^	0.50 ± 0.14 ^aA^

^A–C^ Different capital letters indicate significant difference of the same sample during the storage time (*p* < 0.05). ^a,b^ Different lower-case letters indicate significant difference between samples in the same day of storage (*p* < 0.05).

## Data Availability

The original contributions presented in this study are included in the article. Further inquiries can be directed to the corresponding author.
